# CANNABIS USE IN OLDER ADULTS: EVIDENCE FROM THE HEALTH AND RETIREMENT STUDY

**DOI:** 10.1093/geroni/igad104.0037

**Published:** 2023-12-21

**Authors:** Divya Bhagianadh

**Affiliations:** Rutgers University, Newark, New Jersey, United States

## Abstract

Cannabis use among persons over 50 years old has tripled since 2000. Some studies have found negative outcomes associated with increasing use of cannabis use among older adults such as higher rates of injury and emergency department use. Other studies have shown improvements in pain, sleep and other outcomes such as self-reported health and labor force participation. In this study, we identify individual determinants and outcomes related to cannabis use among adults aged 50+ sourcing data from the 2018 cannabis module of the Health Retirement Survey and constructing a longitudinal panel by linking this data with the respondents’ 2016 and 2020 data. Among the 1,372 respondents, 10% reported past year use. Among these past year users, 35% were persistent, lifelong users and nearly 25% used solely for medical purposes. Multivariate logistic regression controlling for demographics, health and functional status and individual fixed effects showed that those who reported pain in the last wave had higher odds of reporting cannabis use (OR 1.70, p-value-0.03). Troubled sleep was associated with higher odds of cannabis use (significant at 10%). We did not see any effect of cannabis use on pain in later years or subsequent healthcare utilization (hospital or doctor visits). Our results reflect a nationally representative survey of cannabis use among aging Americans and affirms how motives and outcomes of cannabis use are age sensitive.

